# Evaluation of the effect of GSK-3β on liver cancer based on the PI3K/AKT pathway

**DOI:** 10.3389/fcell.2024.1431423

**Published:** 2024-08-02

**Authors:** Jiageng Guo, Xinya Jiang, Jing Lian, Huaying Li, Fan Zhang, Jinling Xie, Jiagang Deng, Xiaotao Hou, Zhengcai Du, Erwei Hao

**Affiliations:** ^1^ Guangxi Key Laboratory of Efficacy Study on Chinese Materia Medica, Guangxi University of Chinese Medicine, Nanning, China; ^2^ Guangxi Collaborative Innovation Center of Study on Functional Ingredients of Agricultural Residues, Guangxi University of Chinese Medicine, Nanning, China; ^3^ Guangxi Key Laboratory of TCM Formulas Theory and Transformation for Damp Diseases, Guangxi University of Chinese Medicine, Nanning, China; ^4^ Ruikang Hospital Affiliated to Guangxi University of Chinese Medicine, Nanning, China

**Keywords:** PI3K/Akt pathway, GSK-3β, liver cancer, regulatory mechanism, signal transduction

## Abstract

The PI3K/AKT/GSK-3β signaling pathway plays a pivotal role in numerous physiological and pathological processes, including cell proliferation, apoptosis, differentiation, and metabolic regulation. Aberrant activation of the PI3K/AKT pathway is intricately linked to development of tumor. GSK-3β, belonging to the serine/threonine protein kinase family, is crucial in the pathogenesis of liver cancer. As a key rate-limiting enzyme in the glucose metabolism pathway, GSK-3β significantly impacts the growth, proliferation, metastasis, and apoptosis of liver cancer cells. It is also implicated in chemotherapy resistance. Elevated expression of GSK-3β diminishes the sensitivity of liver cancer cells to chemotherapeutic agents, thereby playing a substantial role in the development of drug resistance. Consequently, targeting of GSK-3β, particularly within the PI3K/AKT signaling pathway, is regarded as a promising therapeutic strategy for liver cancer. The precise identification and subsequent modulation of this pathway represent a substantial potential for innovative clinical interventions in the management of liver cancer.

## 1 Introduction

Hepatocellular carcinoma (HCC), commonly known as liver cancer, is a malignancy originating from hepatocytes, the primary liver cells. This cancer is notably characterized by its highly invasive and aggressive nature (S et al., 2021). Early diagnosis of HCC is frequently hindered by the absence of conspicuous symptoms during its initial stages. Consequently, this delay often results in missed opportunities for patients to undergo potentially curative treatments such as liver resection and liver transplantation. Despite with radical resection, the prognosis for HCC patients remains largely unfavorable. This is primarily attributed to the high rates of metastasis, spread, and recurrence that are characteristic of liver cancer. Subsequent to liver resection, the recurrence rates for HCC patients at the 3-year and 5-year marks are remarkably elevated, reaching 50% and 70%, correspondingly (ZM et al., 2022). Notably, HCC currently ranks as the third leading cause of cancer-related mortality (M et al., 2018). In the treatment of advanced liver cancer, doxorubicin is commonly utilized. Initial studies reported a response rate of 79% when doxorubicin was used as a monotherapy. However, its clinical efficacy has been found to be limited, evidenced by a response rate of less than 20% and a lack of significant survival benefit. Additionally, statistical data reveals that the 5-year survival rate for patients with HCC in my country is as low as 12% (R et al., 2018).

**FIGURE 1:**
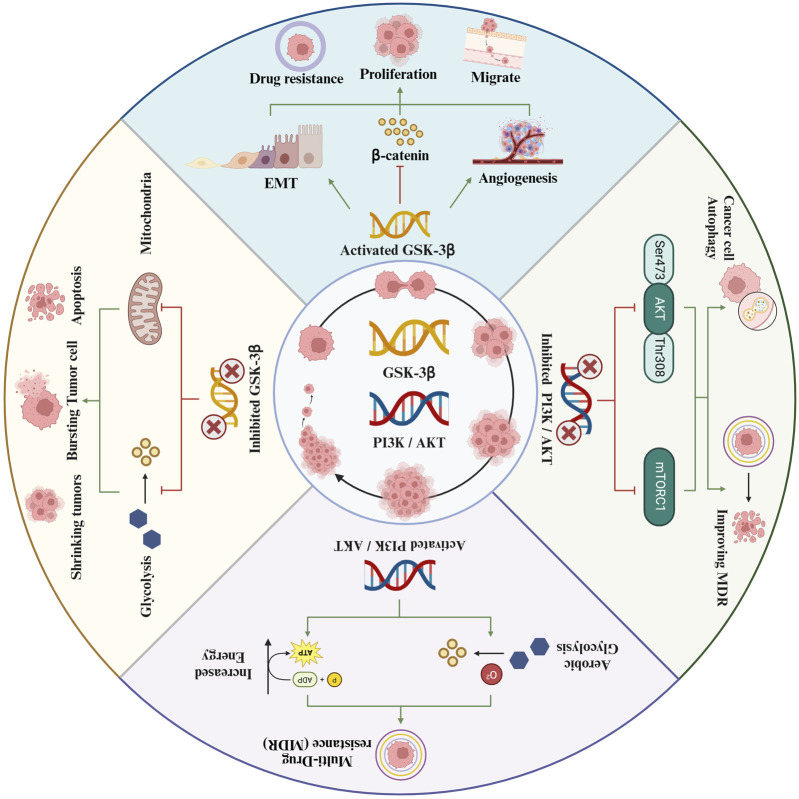
Evaluation of the effect of GSK-3β on liver cancer based on the Pl3K/AKT pathway.

The PI3K/AKT signaling pathway plays a crucial role in tumor growth and progression. It is integral to a multitude of cellular functions and metabolic processes. The pathway’s involvement in cancer cell proliferation, apoptosis, cell cycle regulation, angiogenesis, invasion, metastasis, and therapy resistance has been thoroughly established. Consequently, researchers are increasingly concentrating on the development of targeted therapies that disrupt key components of the PI3K pathway. This strategic focus has led to the emergence of various targeted inhibitors, which have demonstrated promising results in the treatment of cancer (Li et al., 2018). Therefore, exploring the impact of the PI3K/AKT pathway on GSK-3β, and its subsequent effects on the initiation and progression of HCC, offers a novel and promising avenue for future therapeutic interventions. This investigation could unveil critical insights into the molecular mechanisms underpinning HCC and potentially lead to the development of more targeted and effective treatments (Figure 1).

## 2 The biological significance of PI3K/AKT pathway in tumor cells

The PI3K/AKT pathway, a critical cellular signaling pathway, encompasses key proteins such as PI3K, AKT, among others. Its central role in cell signal transduction has led to substantial research interest in recent years. The pathway’s involvement in a wide range of cellular processes, including growth, proliferation, differentiation, motility, survival, and intracellular trafficking, underscores its importance in the development of cancer. Notably, significant progress has been made in identifying components of this pathway, investigating aberrant signaling, and understanding the onset of proliferation disorders due to mutations in this signaling cascade (V et al., 2016). The PI3K/AKT pathway is instrumental in the regulation of fundamental biological processes within cells. It comprises a series of kinase cascades and exerts influence on cellular functions through the orchestration of specific kinases, phosphatases, and regulatory proteins. These components collectively play a pivotal role in phosphorylation and dephosphorylation processes, which are critical for various cellular signaling mechanisms (Steelman et al., 2011), aberrant activation of the PI3K pathway has been observed in a diverse array of malignancies, underscoring its extensive role in cancer development. Specifically, in liver cancer, PI3K has been identified as playing a crucial role, particularly in enhancing the migration and invasion capabilities of liver cancer cells. Consequently, the investigation into the expression and activity of this pathway in tumor cells is of paramount importance, as it holds potential for developing targeted cancer therapies.

## 3 Structure and function of PI3K

PI3K, a lipid kinase, exhibits diversity in its structure, substrate specificity, and mechanism of action, leading to its classification into three distinct types: PI3KⅠ, PI3KⅡ, and PI3KⅢ. Each class plays a unique role, mediating a variety of cellular processes by regulating different phosphoinositides and exhibiting distinct patterns of cellular localization. This classification is pivotal in understanding the multifaceted functions of PI3K in cellular signaling pathways (S et al., 2022).

The activation of PI3KⅠ molecules is highly correlated with tumor development, making it the primary focus of scientific research. Currently, PI3KⅠ is the most extensively investigated molecule in this field. PI3KⅠ has two subtypes, IA and IB. IA is composed of the regulatory subunit p85 (p85α, p85β, p85γ) and the catalytic subunit p110 (p110α, p110β, p110δ, p110γ), forming a heterodimer structure (K and CV, 2022). When various activating stimuli impact PI3K (activation of PI3K ⅠA is primarily induced by receptor tyrosine kinases, growth factors, etc., whereas G protein-coupled receptors activate PI3K ⅠB), the catalytic subunit p110 triggers the activation of the regulatory subunit p85. Consequently, p85 solicits PI3K to adhere to the cellular membrane (JP et al., 2019), phosphatidylinositol 4,5-bisphosphate (PIP2) undergoes phosphorylation at its 30 hydroxyl groups, resulting in phosphatidylinositol 3,4,5-trisphosphate (PIP3) formation. This process is instrumental in activating Akt through the activation of PI3K.

However, the activity of PIP3 is modulated by the tumor suppressor phosphatase and tensin homolog (PTEN). PTEN binds to PIP3 and catalyzes its dephosphorylation, converting it back into PIP2. This action of PTEN is crucial as it serves to prevent the overactivation of PIP3. Consequently, PTEN plays an indispensable role in inhibiting the activation of PIP3, thereby maintaining regulatory control over essential cellular functions (B and R, 2022). When PIP3 is activated, it performs a critical role in recruiting Akt to the cell membrane. Upon its activation, Akt translocates to both the cytoplasm and nucleus, where it regulates a variety of signaling pathways, including mTOR, GSK-3β, FOXO, among others. These downstream effectors are integral in controlling key physiological processes, such as cell growth, proliferation, survival, and metabolism. Understanding the interaction and regulation of these molecules provides insight into the complex mechanisms governing cellular behavior.

## 4 Structural functions of AKT

AKT is a pivotal component in cellular signaling, orchestrating a wide array of cellular processes. However, the clinical application of Akt inhibitors is complicated by their high toxicity. Additionally, the three subtypes of AKT—each with distinct functions—add complexity to their therapeutic targeting. Consequently, the molecular mechanisms related to these AKT subtypes continue to be an active area of research (Bao et al., 2021). Recent studies have revealed that AKT, referred to as protein kinase B (PKB), operates as a serine/threonine kinase. This enzyme is encoded by three distinct genes, giving rise to three unique AKT subtypes: AKT1/PKBα, AKT2/PKBβ, and AKT3/PKBγ (I, 2020).

Akt1 is ubiquitously distributed across various tissues, underscoring its importance in a multitude of physiological processes. In contrast, Akt2 predominantly exists in tissues characterized by high insulin sensitivity, highlighting its vital role in insulin signaling and glucose metabolism. Akt3, meanwhile, is primarily expressed in the brain and testis, signifying its specialized involvement in neuronal and reproductive functions. These distinct tissue-specific distribution patterns of Akt isoforms emphasize their diverse functional roles in varying physiological contexts. Previous research has elucidated the structural composition of AKT, revealing a pleckstrin homology (PH) domain at its N-terminal, a catalytic domain in the central region, and a regulatory region at the C-terminal. The PH domain of Akt binds to PIP2 and PIP3 metabolites, a critical step in its recruitment to the cell membrane. Upon localization, AKT exposes its activation domain (Thr308) and hydrophobic motif (Ser473), undergoing phosphorylation mediated by phosphoinositide-dependent kinase 1 (PDK1) and the mTOR complex 2 (mTORC2) (PJ et al., 2022), causes complete activation of AKT.

AKT is an important protein that plays a critical role in various cellular processes, including cell proliferation, cell cycle regulation, apoptosis, autophagy, and angiogenesis. Its complete activation is linked to the development and progression of tumors. Phosphorylation of Akt can lead to the activation of signaling pathways in liver cancer cells (Aaron and Brendan, 2021), Akt activity is typically tightly regulated in normal cell functions, but in liver cancer cells, elevated levels of phosphorylation lead to increased activity. This heightened phosphorylation can result from various mechanisms, including activation of cell surface receptors, mutated signaling proteins, and abnormal intracellular signaling. Phosphorylation of Akt activates multiple signaling pathways, such as activating the PI3K/Akt/mTOR pathway (Antonino et al., 2023), which promotes protein synthesis, cell growth and proliferation; activating GSK-3β in the PI3K/Akt/GSK-3β pathway promotes the occurrence of liver cancer (H et al., 2023), thereby promoting the proliferation of liver cancer cells, inhibiting apoptosis, increasing cell migration and invasion capabilities, and promoting tumor angiogenesis. Consequently, Akt phosphorylation plays a crucial role in the initiation and progression of liver cancer. Researchers are actively developing anti-tumor drugs targeting the Akt signaling pathway to suppress liver cancer cell proliferation and survival. These drugs could offer novel treatment approaches for liver cancer patients, potentially improving treatment outcomes.

## 5 Relevance of glycogen synthase kinase 3β (GSK-3β) in tumor cells

Glycogen synthase kinase 3β (GSK-3β), a prominent serine/threonine protein kinase, is recognized as an essential substrate of AKT. Ubiquitously present in eukaryotic cells, GSK-3β is integral to a range of cellular processes (SA et al., 2018). GSK-3β, a rate-limiting enzyme of glycogen synthase kinase, has been found to be involved in various physiological processes such as cell proliferation, differentiation, and apoptosis. It is also associated with the occurrence of diseases including tumors, inflammation, and Alzheimer’s disease. A critical function of GSK-3β is the regulation of blood sugar homeostasis, achieved through the phosphorylation of various endogenous substrates. These substrates, encompassing a range of proteins and transcription factors, are pivotal in metabolic processes. Specifically, they play significant roles in the growth, proliferation, apoptosis, migration, and metastasis of tumor cells. Thus, GSK-3β emerges as a vital player in these extensive and interconnected biological processes (A and KN, 2020).

Research into therapies targeting GSK-3β has produced divergent outcomes. Studies have revealed that both inhibition and activation of GSK-3β can significantly impact tumor initiation and progression. Empirical investigations have confirmed GSK-3β′s involvement in a wide spectrum of malignancies, including prostate, colorectal, gastric, pancreatic, liver, ovarian, osteosarcoma, leukemia, non-small cell lung, bladder, thyroid, glioma, kidney cancers, and other malignant tumors. Cellular and tissue analyses consistently demonstrate the abundant expression of GSK-3β in these cancers, underscoring its potential as a key target in cancer therapy (R, 2010), This shows that GSK-3β is significantly related to the occurrence and development of these tumors. Interestingly, several investigations have demonstrated that GSK-3β can impede the progression, expansion, and spread of cancerous tumors like cutaneous carcinoma, oral carcinoma, laryngeal carcinoma, esophageal carcinoma, mammary carcinoma, salivary gland carcinoma, nasopharyngeal carcinoma, and melanoma (R, 2010), low expression of GSK-3β was detected in cells and tissues of these tumors.

GSK-3β is critically significant in both the initiation and progression of tumors, playing a pivotal role in the expansion, reproduction, spread, and programmed cell death of cancer cells. Furthermore, its association with tumor resistance to chemotherapy underscores its importance as a therapeutic target (T-Y, 2013). GSK-3β has emerged as a valuable asset in clinical oncology, serving as a crucial prognostic marker for tumors (James et al., 2014). Its role extends beyond prognostication to offering significant therapeutic potential in inhibiting tumor growth and metastasis. Additionally, GSK-3β plays a role in modulating resistance to both chemotherapy and radiotherapy. Exploiting GSK3β′s multifaceted role in these areas is increasingly seen as a promising strategy for enhancing the efficacy of cancer treatments, potentially leading to improved patient outcomes (JA et al., 2014).

The scarcity of consolidated and accessible summaries of research on the impact of various drugs and compounds targeting GSK-3β within the PI3K/Akt pathway, specifically in the context of liver cancer, has posed challenges in information retrieval and synthesis. This article seeks to bridge this gap by systematically collecting and reviewing pertinent research literature. The goal is to provide a comprehensive overview of current findings, thereby facilitating a clearer understanding of the therapeutic potential and limitations of these interventions in liver cancer treatment.

## 6 The role of GSK-3 in hepatocellular carcinoma

Glycogen synthase kinase 3 (GSK-3) is a serine/threonine kinase that plays a pivotal role in regulating various cellular processes, including metabolism, cell proliferation, differentiation, and apoptosis. GSK-3 exists in two isoforms: GSK-3α and GSK-3β. Despite their structural similarities, these isoforms have distinct functions and regulatory mechanisms.

GSK-3α and GSK-3β are highly conserved enzymes that differ slightly in their molecular weight and functional domains. GSK-3α has a molecular weight of approximately 51 kDa, while GSK-3β is about 47 kDa. Both isoforms are ubiquitously expressed and play crucial roles in various signaling pathways. GSK-3α is primarily involved in cellular metabolism and insulin signaling, while GSK-3β is more critically implicated in Wnt/β-catenin signaling, cell cycle regulation, and apoptosis (Doble and Woodgett, 2003; Jope and Johnson, 2004).

The PI3K/AKT signaling pathway is a central regulator of cell survival, growth, and proliferation. It is frequently dysregulated in many cancers, including HCC. Both GSK-3α and GSK-3β are downstream targets of the PI3K/AKT pathway and are involved in its feedback regulation. Upon activation by PI3K, AKT phosphorylates and inactivates GSK-3β, which in turn affects various downstream targets involved in cell proliferation and survival (Cross et al., 1995; Cohen et al., 2001). This regulation is crucial for maintaining cellular homeostasis and preventing uncontrolled cell growth.

While GSK-3β has been extensively studied in the context of HCC, GSK-3α also plays significant roles in the disease’s progression. GSK-3α is heavily involved in the regulation of glucose metabolism, which is a hallmark of cancer cells, including HCC. It modulates insulin signaling and glycogen synthesis, which are critical for cancer cell energy supply and growth (Cohen et al., 2001; Medina and Wandosell, 2011). Similar to GSK-3β, GSK-3α can influence cell proliferation. Studies have shown that inhibition of GSK-3α can reduce the growth of HCC cells, indicating its potential as a therapeutic target (Emma et al., 2020). Additionally, GSK-3α is involved in various signaling pathways beyond PI3K/AKT, including the regulation of NF-κB, which plays a role in inflammation and cancer progression (Parajuli et al., 2011).

In HCC, the dysregulation of GSK-3β has been shown to contribute significantly to tumorigenesis. GSK-3β promotes the proliferation of HCC cells by modulating the activity of various cell cycle proteins, including cyclin D1 and c-Myc (Fang et al., 2007). The inhibition of GSK-3β has been demonstrated to reduce the proliferation of HCC cells, indicating its potential as a therapeutic target. GSK-3β influences the progression of the cell cycle by regulating proteins that control the G1/S transition. This regulation ensures that cells only proliferate when appropriate signals are present, preventing unregulated cell division that can lead to cancer (Zhang et al., 2009). Furthermore, GSK-3β has anti-apoptotic effects in HCC cells, helping them evade programmed cell death. It phosphorylates and inactivates several pro-apoptotic factors, thereby enhancing cell survival and contributing to the resistance of HCC cells to apoptosis-inducing therapies (C B. et al., 2022). Additionally, GSK-3β plays a role in shaping the tumor microenvironment by affecting the secretion of cytokines and growth factors that promote angiogenesis and immune evasion, facilitating tumor growth and metastasis (Massagué, 2004).

GSK-3, particularly GSK-3β, plays a crucial role in the development and progression of hepatocellular carcinoma through its regulation of the PI3K/AKT pathway and other cellular processes. Its involvement in cell proliferation, survival, and the tumor microenvironment underscores its potential as a therapeutic target. Additionally, GSK-3α′s role in metabolic regulation and signal transduction further contributes to the complex landscape of HCC pathology. Ongoing research will further elucidate the therapeutic potential of targeting GSK-3 in HCC, offering hope for improved treatment strategies for this challenging malignancy.

## 7 Effects of intervening GSK-3β on liver cancer

### 7.1 Activating GSK-3β promotes the occurrence of liver cancer

In recent research, various factors such as surface proteins, glycoproteins, peroxisome proliferator-activated receptors (PPARs), and non-coding RNAs have been identified as contributing to the development of liver cancer. These elements, notably, have been shown to activate GSK-3β, which in turn promotes the proliferation and metastasis of liver cancer cells. Consequently, deciphering the mechanisms by which GSK-3β activation fosters liver cancer progression is essential for devising effective preventive strategies. Continued research into the activation pathways of GSK-3β holds promise for the development of novel interventions aimed at mitigating the incidence of liver cancer.

#### 7.1.1 CD58 activates GSK-3β to promote hepatocellular carcinoma growth

CD58, a cell surface protein known for its extensive glycosylation, exhibits widespread expression across a range of hematopoietic and non-hematopoietic cells. It exists in two primary forms: as a type I transmembrane protein or as a glycosylphosphatidylinositol (GPI)-anchored protein. (Y et al., 2021). CD58 is also present in a soluble form (sCD58) in human serum, urine, and cell culture supernatants. Experimental studies have underscored the importance of CD58 in the context of tumors (B et al., 2023). These findings suggest that CD58, both in its membrane-bound and soluble forms, may play a significant role in tumor biology, potentially influencing tumor development, progression, and interaction with the immune system. Extensive research has established that a significant number of hematological malignancies are characterized by a marked decrease or even complete absence of CD58 expression. In contrast, certain solid tumors, such as gastric cancer, colorectal malignancies, and malignant gliomas, have been found to exhibit a substantial upregulation of CD58. This dichotomy in CD58 expression patterns across various cancer types underscores its complex role in tumor biology and potentially indicates different mechanisms of tumor progression and immune system interaction (S et al., 2015). The activation of CD58 may contribute to the upregulation of the Wnt/β-catenin pathway, a process that is implicated in promoting tumor self-renewal capabilities. Recent investigations have revealed a significant upregulation of CD58 in HCC tissues (D L. et al., 2021). However, the role and underlying mechanisms of CD58 in HCC remain unclear.

Wang Chuanzheng and colleagues (C et al., 2023) have conducted experiments demonstrating the significant expression of CD58 in liver cancer. Employing immunofluorescence staining and Western blot techniques, they conducted both quantitative and qualitative analyses of CD58 expression. Their research findings have established a correlation between increased CD58 expression and various clinicopathological features, including tumor size, differentiation level, and postoperative prognosis in liver cancer patients. Moreover, multiple experimental studies have consistently shown that CD58, along with its soluble variant sCD58, can activate the PI3K/AKT signaling pathway. This activation is achieved by binding to specific proteins in the cell membrane, subsequently stimulating the GSK-3β target. Such stimulation promotes the growth, migration, invasion, and maintenance of hepatocellular carcinoma stem cell characteristics. Further investigation into serum samples from hepatocellular carcinoma patients revealed significantly elevated levels of sCD58 compared to healthy individuals, suggesting its potential role in the development of hepatocellular carcinoma. Both *in vivo* and *in vitro* assessments have conclusively demonstrated CD58’s functional role in enhancing the proliferation of liver cancer cells.

The findings of the study elucidate that the elevated expression of CD58 is intimately associated with the activation of the GSK-3β target. This suggests that the CD58-mediated pathway plays a pivotal role in the proliferation and progression of HCC cells. Consequently, CD58 and sCD58 have the potential to serve as significant prognostic indicators for hepatocellular carcinoma, offering new avenues for exploring innovative approaches to treating this form of cancer.

#### 7.1.2 Regulation of the PI3K/AKT/GSK-3β pathway by non-coding RNAs in liver cancer

Liver cancer, predominantly hepatocellular carcinoma (HCC), remains one of the leading causes of cancer-related mortality worldwide. The PI3K/AKT/GSK-3β signaling pathway is crucial for regulating cell survival, proliferation, and metabolism, and its dysregulation is a hallmark of HCC. Non-coding RNAs (ncRNAs), particularly microRNAs (miRNAs) and long non-coding RNAs (lncRNAs), have emerged as critical regulators of this pathway, influencing liver cancer development and progression.

MicroRNAs (miRNAs) are a class of small, non-coding RNAs that play a pivotal role in gene regulation. They have garnered significant attention for their ability to regulate the expression of a broad spectrum of genes, including both tumor suppressor genes and oncogenes (HM et al., 2015). There is an accumulating body of research underscoring the critical involvement of miRNAs in the initiation and progression of tumors. Specifically, abnormal expression patterns of certain miRNAs, such as miR-96, have been closely linked to liver cancer. Numerous studies have reported a significant upregulation of miR-96 in HCC. Importantly, strategies aimed at inhibiting the activity of miR-96 have shown promise in slowing down HCC progression. Moreover, aberrant expression of miR-96 has also been identified in other malignancies, including colorectal cancer, osteosarcoma, and prostate cancer, suggesting its broader oncogenic role across different cancer types. (X F. et al., 2018; YH et al., 2017; Q et al., 2018; AL et al., 2015). Wang TH et al. (TH et al., 2016)found that miR-96 functions as a tumor miRNA in HCC. Inhibition of miR-96 resulted in reduced HCC cell proliferation and migration. Baik SH et al. (SH et al., 2016)confirmed the role of miR-96 in promoting tumor growth by implanting miR-96-overexpressing HepG2 cells in a xenograft model. Chen Y et al. (Y et al., 2015) discovered that elevated levels of miR-96 in the serum were strongly correlated with increased rates of metastasis to the lymph nodes and improved overall survival in patients with HCC.

The exact molecular mechanism by which miR-96 affects the progression of HCC involves the activation of the PI3K/AKT/GSK-3β signaling pathway. Yang Nanmu et al. (N et al., 2019) utilized qRT-PCR and Western blot to detect the expression of mRNA and protein respectively. Their study revealed that miR-96 plays a pivotal role in activating the PI3K/AKT/GSK-3β signaling pathway in hepatocellular carcinoma. Specifically, the activation of miR-96 induces AKT phosphorylation, which in turn promotes the phosphorylation of GSK-3β. This phosphorylation diminishes GSK-3β′s ability to degrade β-catenin, leading to its accumulation in the cytoplasm and subsequent translocation into the nucleus. Once in the nucleus, β-catenin enhances the activation of downstream genes, contributing to tumor progression. (Eshelman and Yochum, 2016).

One of the most studied miRNAs in HCC is miR-21. This miRNA is frequently overexpressed in HCC and targets PTEN, a crucial tumor suppressor that negatively regulates the PI3K/AKT pathway. PTEN functions by dephosphorylating PIP3 back to PIP2, thereby inhibiting the AKT signaling cascade. The downregulation of PTEN by miR-21 leads to enhanced PI3K/AKT signaling, resulting in increased cell proliferation, migration, and invasion. This promotes tumor growth and metastasis, underscoring miR-21’s role as an oncogene in liver cancer (Meng et al., 2007; Liu et al., 2010).

Similarly, miR-221 and miR-222 are upregulated in HCC and target both PTEN and TIMP3. While PTEN acts as a direct inhibitor of the PI3K/AKT pathway, TIMP3 controls extracellular matrix degradation and cell proliferation signals, indirectly influencing the same pathway. The suppression of PTEN and TIMP3 by miR-221/222 leads to the activation of the PI3K/AKT pathway, which facilitates tumorigenesis and provides resistance to apoptosis. These miRNAs enhance the malignancy of HCC by promoting cancer cell survival and proliferation, as evidenced by experimental models and clinical samples (Fornari et al., 2008; Garofalo et al., 2009).

miR-29 is another critical miRNA that targets GSK-3β, a serine/threonine kinase involved in multiple cellular processes, including the regulation of the Wnt/β-catenin signaling pathway. GSK-3β typically phosphorylates and destabilizes β-catenin, preventing it from translocating to the nucleus and activating oncogenic transcription programs. By downregulating GSK-3β, miR-29 prevents the phosphorylation and degradation of β-catenin, leading to its accumulation and activation. This enhances cell proliferation and survival, contributing to the aggressive nature of HCC (Cao et al., 2020; Qian et al., 2023).

In addition to miRNAs, lncRNAs play a significant role in modulating the PI3K/AKT/GSK-3β pathway. HULC (Highly Upregulated in Liver Cancer) is a notable lncRNA that acts as a molecular sponge for miR-372, which directly targets the PI3K/AKT pathway. By binding to miR-372, HULC prevents miR-372 from interacting with its mRNA targets, indirectly activating the PI3K/AKT pathway. This leads to increased cell proliferation and tumorigenicity, highlighting the oncogenic potential of HULC in liver cancer (Wang et al., 2010).

MALAT1 (Metastasis-Associated Lung Adenocarcinoma Transcript 1) is another lncRNA highly expressed in HCC. MALAT1 interacts with various components of the PI3K/AKT pathway, modulating gene expression by acting as a scaffold for protein complexes and influencing chromatin dynamics. This lncRNA promotes cell growth, metastasis, and inhibits apoptosis in liver cancer cells by modulating the PI3K/AKT signaling pathway. High MALAT1 expression correlates with poor prognosis in HCC patients, making it a critical biomarker and potential target for therapeutic intervention (Gutschner et al., 2013; Yu et al., 2017).

Non-coding RNAs, including miRNAs and lncRNAs, are pivotal regulators of the PI3K/AKT/GSK-3β signaling pathway in liver cancer. They modulate key components of this pathway, influencing tumor growth, metastasis, and resistance to apoptosis. Understanding these regulatory mechanisms provides insights into potential therapeutic targets for HCC. Future research should focus on elucidating the detailed molecular interactions and therapeutic implications of ncRNAs in liver cancer.

#### 7.1.3 sCLU activates PI3K/AKT/GSK-3β to promote the development of hepatocellular carcinoma

Clusterin (CLU), also known as Apo-J, is a disulfide-linked heterodimeric glycoprotein highly conserved across species. It is ubiquitously present in various tissues and body fluids, playing multifaceted roles in several physiological processes. These processes include lipid transport, apoptosis, the complement cascade, DNA repair, and cell adhesion. Upon secretion, CLU matures into its sulfated form, sCLU, a glycoprotein with a molecular weight ranging between 70 and 80 kDa. This mature form is synthesized from a single-copy gene located at the chromosomal position 8p21. Predominantly localized in the cytoplasm, sCLU is involved in a diverse array of biological functions, encompassing lipid transport, senescence, the complement cascade, membrane recycling, cell adhesion, and programmed cell death (M et al., 2012). The sCLU protein plays a crucial role in inhibiting cell death, promoting the growth of new blood vessels, and supporting the spread of tumors. It is frequently observed to be overexpressed in various types of cancerous tumors (X et al., 2014). sCLU, being highly efficient in both diagnosing and predicting hepatocellular carcinoma, has gained significant recognition as a biomarker.

Numerous studies have shed light on the role of sCLU in the progression of HCC. These investigations suggest that sCLU functions as a regulatory factor in the AKT/GSK-3β signaling pathway. Such a role of sCLU has been linked to its influence on the *in vivo* growth of HCC. Consequently, sCLU’s involvement is observed to trigger specific responses in HCC cells towards sorafenib/AKT, which include the development of chemotherapy resistance, tumor metastasis, and enhanced tumor growth. (W et al., 2017). Zheng Wenjie et al. (W et al., 2020) conducted a study on liver specimens obtained from 72 patients with HCC who underwent liver resection. This research revealed that sCLU possesses the capability to activate the AKT/GSK-3β/β-catenin signaling pathway. This activation by sCLU was found to lead to chemotherapy resistance, metastasis, and an unfavorable prognosis in patients diagnosed with HCC.

In summary, the aforementioned studies collectively demonstrate that sCLU plays a pivotal role in stimulating the PI3K/AKT/GSK-3β signaling pathway, a process that significantly contributes to the progression and exacerbation of malignant characteristics in HCC. This enhanced understanding of sCLU’s role in HCC underscores its potential as a promising molecular target.

#### 7.1.4 PPARδ activates PI3K/AKT/GSK-3β to promote the proliferation and invasion of liver cancer cells

Peroxisome proliferator-activated receptor (PPAR) belongs to the superfamily of ligand-activated nuclear transcription factors. PPARα (PPARA), PPARδ/β (PPARD), and PPARγ (PPARG) exist in various organs and contribute to diverse energy metabolism mechanisms, holding substantial significance in the advancement of tumors (D M. et al., 2021). The expression of PPARδ can be observed in various parts of the human body and its expression levels vary in different types of cancer, including colorectal, gastric, and prostate cancer. PPARδ has the potential to act as either an oncogene or a tumor suppressor gene, and the specific mechanisms through which it exerts these effects are diverse (Y et al., 2019). The activation or heightened expression of PPARδ has been increasingly recognized as playing a pivotal role in cell proliferation and tumor growth, with numerous studies substantiating this association. Additionally, PPARδ has been identified as a critical regulatory factor in tumor metastasis. Its role is particularly evident in promoting key processes such as angiogenesis, epithelial-mesenchymal transition (EMT), invasion, and migration. The involvement of PPARδ in these diverse mechanisms not only underlines its importance in tumor progression but also highlights its potential as a promising target for therapeutic interventions aimed at combating cancer. (KD et al., 2019).

In the study, HanWei et al. (W et al., 2022) employed a range of techniques to evaluate RNA and protein expression levels, including analysis of The Cancer Genome Atlas (TCGA) and Gene Expression Omnibus (GEO) transcriptome data, real-time quantitative polymerase chain reaction (qRT-PCR), Western blotting, and immunohistochemistry. Additional assessments were conducted using the Cell Counting Kit-8 (CCK8) and 5-Ethynyl-2ʹ-deoxyuridine (EdU) assays. The study found that PPARδ expression was notably higher in HCC tissues compared to normal liver tissues. Immunohistochemistry analysis further confirmed PPARδ′s overexpression at the protein level in HCC tissues. Importantly, PPARδ′s expression showed significant clinical relevance with respect to TNM stage and pathological grade in HCC. Kaplan-Meier (K-M) survival curves, derived from Overall Survival (OS), Disease-Specific Survival (DSS), Progression-Free Survival (PFS), and Cox regression analysis, indicated a considerably worse prognosis for patients with high PPARδ expression. Additionally, HCC cell lines were observed to express high levels of PPARδ. Through cellular function experiments, the study verified that PPARδ could stimulate liver cancer cell proliferation, migration, and angiogenesis by activating the PI3K/AKT/GSK-3β signaling pathway.

#### 7.1.5 PDCD6 promotes liver cancer cell proliferation and metastasis through the AKT/GSK-3β pathway

Programmed cell death 6 (PDCD6), also known as apoptosis-linked gene-2 (ALG-2), is a calcium-binding protein ubiquitously present in various cells across the body. A number of studies have been conducted to assess the expression levels of PDCD6 in tumor tissues and cell lines derived from clinical samples. These investigations have consistently revealed that PDCD6 expression varies among different types of tumors, indicating its potential role in tumorigenesis. Furthermore, research utilizing animal models, particularly in rats, has shown that PDCD6 expression is significantly higher in liver cancer tissues compared to normal liver tissues (JM et al., 2003). According to a study, PDCD6 is abundantly present in both human lung cancer tissues and metastatic cells of ovarian cancer (D et al., 2012). Additionally, the study reveals that the expression levels of PDCD6 are comparatively lower in non-small cell lung cancer and gastric cancer (YQ et al., 2012).

Despite the above observations, PDCD6 has not been identified as an oncogene or tumor suppressor in HCC. Wen Shiyuan et al. (SY et al., 2023), conducted a comprehensive investigation into the roles of PDCD6 in human HCC tissues and cancer cell lines. Their initial analysis of The Cancer Genome Atlas (TCGA) database revealed a significant correlation between high PDCD6 expression and poor prognosis in HCC patients, hinting at PDCD6’s crucial role in HCC development. To delve deeper, the research team analyzed PDCD6 expression levels in liver cancer cell lines derived from patients, employing bioinformatics tools and Western blotting techniques. The team also evaluated cell viability and migratory capabilities using the MTT assay and transwell migration assays, respectively. Findings from these experiments demonstrated that high PDCD6 expression markedly enhanced the proliferation, migration, and invasion capabilities of liver cancer cells. Conversely, the inhibition of PDCD6 led to a reduction in cell viability and metastatic potential. Other investigations elucidated that elevated PDCD6 expression in HCC cells activated the AKT pathway, which in turn phosphorylated GSK-3β. This phosphorylation stimulated HCC tumorigenesis. Additionally, the study revealed that PDCD6 could promote HCC cell migration and invasion via the process of EMT. These findings provide valuable insights into the potential mechanisms through which PDCD6 contributes to the onset and progression of hepatocellular carcinoma, suggesting its role as a significant molecular target in HCC research and therapy.

**FIGURE 2:**
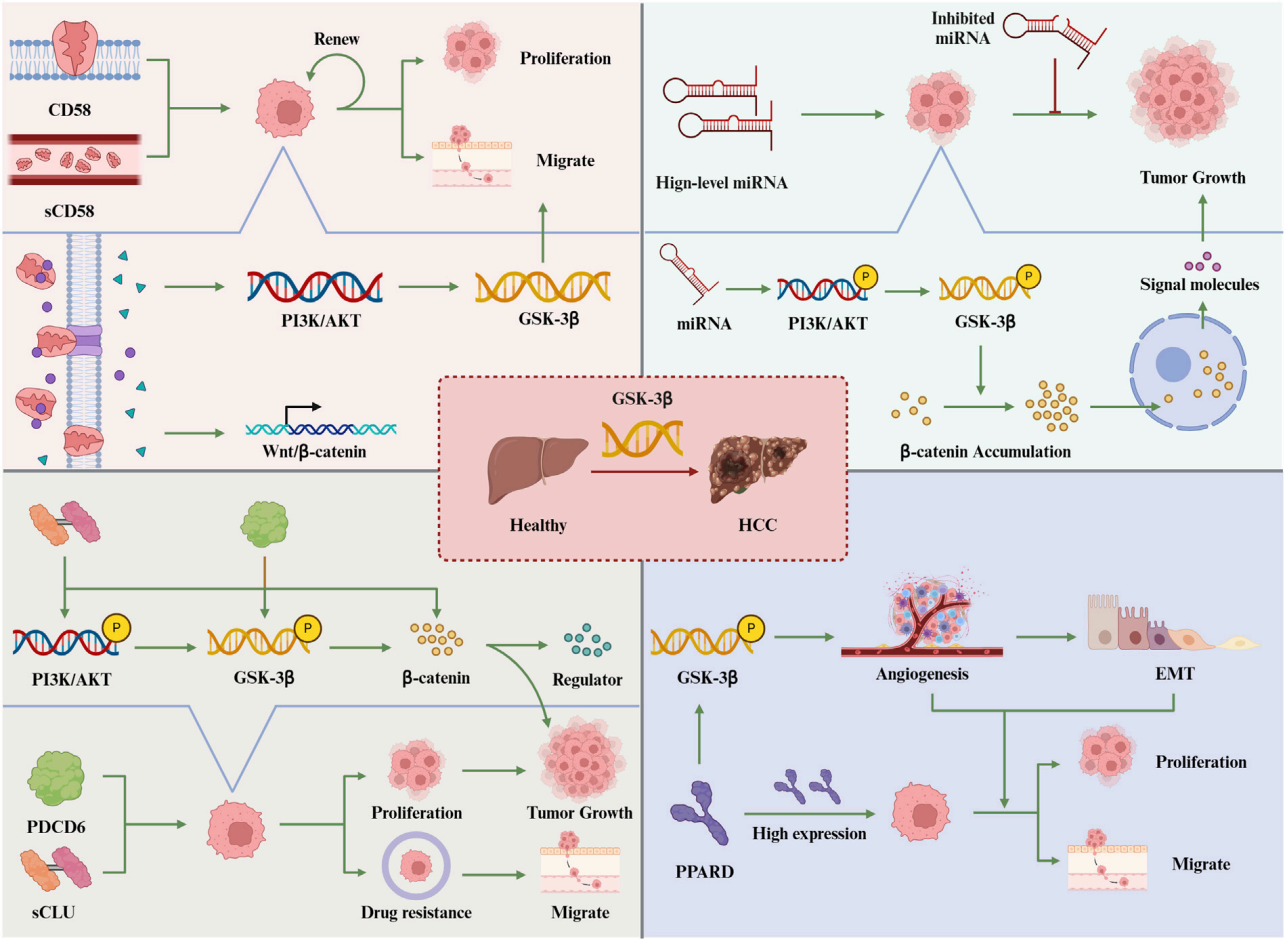
Activating GSK-3β promotes the occurrence of liver cancer.

In summary, PDCD6 plays a pivotal role in promoting tumor growth in HCC, primarily through its modulation of the AKT/GSK-3β signaling pathway. As a key oncogene, PDCD6 influences the levels of various transcription factors, thereby driving the proliferation and metastasis of HCC cells. By governing the AKT/GSK-3β pathway, PDCD6 emerges as a significant contributor to tumor progression in HCC. This understanding positions PDCD6 as a promising target for the development of targeted therapies aimed at inhibiting the progression of HCC (Figure 2).

### 7.2 Inhibiting GSK-3β inhibits the occurrence of liver cancer

The activation of GSK-3β has been identified as a key factor in the development of liver cancer. Inhibiting GSK-3β has been shown to lead to significant metabolic changes in tumor cells, such as a reduction in aerobic glycolysis. Furthermore, GSK-3β inhibition can also induce mitochondrial apoptosis within these cells. These apoptotic effects have been observed to effectively impede the onset and progression of liver cancer.

#### 7.2.1 Inhibiting aerobic glycolysis of tumor cells and promoting apoptosis

Energy metabolism reprogramming represents a critical hallmark of cancer, significantly impacting various cellular processes including cell proliferation, differentiation, and metastasis. Tumor cells exhibit a unique metabolic behavior characterized by altered energy metabolism, even in the presence of ample oxygen. These cells predominantly engage in aerobic glycolysis, where there is an increased production of lactate rather than relying on oxidative phosphorylation for energy. This metabolic shift results in a markedly higher rate of glucose consumption, diverging from the metabolic pathways typically observed in non-cancerous cells (C and L, 2018). Cancer cells often exhibit a preference for aerobic glycolysis, a shift frequently attributed to mitochondrial damage. This increased reliance on glycolysis not only facilitates ATP generation but also significantly influences tumor growth and metastasis. One of the byproducts of heightened glycolysis is lactic acid, which, when produced in excess, surpasses the body’s buffering capacity. This leads to an accumulation of lactic acid, resulting in the acidification of the tumor microenvironment. Such an acidic extracellular milieu is detrimental to the surrounding normal cells, often leading to their death. Additionally, this altered microenvironment contributes to the remodeling of the extracellular matrix (ECM), thereby promoting the EMT process, which is a critical step in cancer cell metastasis, facilitating the invasion and spread of tumor cells (MT et al., 2019).

Aerobic glycolysis, often referred to as the ‘Warburg effect,’ is recognized as a defining feature of cancer metabolism and has been intricately linked to the development of HCC (J et al., 2022). Within this metabolic context, GSK-3β emerges as a crucial enzyme in regulating glucose metabolism in cancer cells. Extensive research has established GSK-3β′s pivotal role in the glycolytic processes of liver cancer cells. Inhibition of GSK-3β activity has been shown to result in reduced glucose uptake, decreased lactate production, and lowered ATP levels in hepatocytes. Moreover, this inhibition also leads to the downregulation of key glycolytic enzymes, suggesting a significant impact on the metabolic pathways critical for tumor cell survival and proliferation (G et al., 2019), Thereby promoting the apoptosis of liver cancer cells.

Salvigenin, a flavonoid compound found in abundance in plants like Pinellia ternata and Skullcap. It has been shown to induce cell cycle arrest and promote apoptosis in colon cancer cells, suggesting its effectiveness in inhibiting tumor growth. Furthermore, in mouse models, Salvigenin has exhibited the ability to enhance anti-tumor immunity and effectively impede the growth of breast cancer (S et al., 2013). However, there are few studies on the role and mechanism of Salvigenin in HCC. Hui Shao et al. (H et al., 2023) conducted a study using various doses of Salvigenin to treat liver cancer. The *in vitro* experiments assessed cell viability, colony formation, cell migration and invasion, apoptosis, as well as glucose uptake and lactate production in HCC cells. The results demonstrated that Salvigenin effectively inhibited the growth, mobility, and infiltration of HCC cells in a concentration-dependent manner. Notably, it also suppressed glycolysis by reducing glucose absorption and lactate production, leading to decreased levels of key glycolytic enzymes HK2, PFK1, and PKM2. Further investigations revealed that Salvigenin impedes the phosphorylation of GSK-3β by inhibiting the PI3K/AKT signaling pathway. Specifically, Salvigenin significantly counteracted the stimulatory effects of the PI3K activator 740Y-P on this pathway.

*In vivo* experiments were conducted using a nude mouse transplanted tumor model treated with Salvigenin. The study evaluated the impact of Salvigenin on HCC cell growth *in vivo*, employing histopathological analysis with HE staining, immunohistochemical staining for Ki67, and TUNEL staining for apoptosis detection. The findings indicated that Salvigenin suppressed the growth of liver cancer cells in nude mice and enhanced apoptosis. Importantly, HE staining showed that Salvigenin did not cause noticeable pathological damage to vital organs in mice. Tumor growth, volume, and weight were effectively reduced in a dose-dependent manner, without significant toxicity to the organs of tumor-bearing mice.

Additionally, the combination of Salvigenin with the chemotherapeutic agent 5-FU was explored. This combination therapy reduced the IC50 value of 5-FU in drug-resistant liver cancer cell lines. Colony formation assays indicated a significant reduction in cell colonies with the combined treatment, compared to 5-FU alone. These findings suggest that Salvigenin can enhance the response of liver cancer cells to chemotherapy and decrease the resistance of drug-resistant liver cancer cells to 5-FU. This effect is primarily attributed to the inhibition of HCC cell proliferation, migration, and invasion via modulation of the PI3K/AKT/GSK-3β pathway. Furthermore, Salvigenin also weakens cellular glycolysis and promotes apoptosis, thus reducing resistance to 5-FU chemotherapy.

Dezocine, is classified as an opioid receptor partial agonist/antagonist (X et al., 2022), has garnered increasing attention as an alternative medication for perioperative pain management (C Z. et al., 2022). This interest is partly due to Dezocine’s comparative advantage in suppressing respiratory depression. Because dezocine’s overall performance in suppressing breathing is stronger than all other drugs on the market, And can alleviate the dependence and addiction caused by the use of morphine (FX et al., 2019). Notably, Dezocine exhibits stronger respiratory suppression compared to other market-available drugs. Furthermore, it has been observed to alleviate the dependency and addiction issues commonly linked with morphine use. Consequently, Dezocine has been increasingly chosen for cancer surgery applications, serving as a crucial analgesic during the perioperative period (DI et al., 2019).

Recent research has shed light on the ability of opioids to influence tumor cells through their interaction with opioid receptors and other cellular receptors. This has led to the hypothesis that opioids can affect tumor cell behavior via multiple mechanisms (DP et al., 2020). In the context of perioperative stress and intervention, these effects are considered to balance each other (JA et al., 2018). Dezocine, commonly used during the perioperative period, has been the subject of limited studies regarding its direct impact on tumor treatment. ZiWen Zhong et al. (ZW et al., 2020) conducted a study to investigate the effects of Dezocine on HepG2, a hepatic cancer cell line. Using the CCK8, wound healing, and transwell assays, the team assessed the viability and migration capabilities of HepG2 cells. Additionally, the aerobic glycolysis of liver cancer cells was evaluated by measuring the extracellular acidification rate (ECAR). The study observed a concentration-dependent influence of Dezocine on HepG2 cells (*p* < 0.01), indicating its potential impact on liver cancer cell viability. In terms of aerobic glycolysis, Dezocine exhibited varying effects, notably by modulating the Akt1-GSK-3β pathway, which in turn altered the activity of glycolytic enzymes HK2 and LDHA.

#### 7.2.2 Promote mitochondrial apoptosis of liver cancer cells and inhibit the development of liver cancer cells

Mitochondria, key organelles in eukaryotic cells, play a vital role in energy production and metabolic regulation. Dysfunctions in these organelles have been consistently linked to the initiation, progression, and invasiveness of tumors (E et al., 2017). Tumor cells, in contrast to normal cells, possess the unique ability to proliferate continuously under favorable conditions. Various research studies have highlighted that high levels of mitochondrial reactive oxygen species (ROS) can induce apoptosis, or programmed cell death, thereby inhibiting tumor formation and growth (K et al., 2014). ROS are molecules characterized by highly reactive oxygen free radicals. Experimental studies have shown that elevated ROS levels can damage the mitochondrial membrane, altering its permeability. This change in membrane permeability leads to a reduction in membrane potential, primarily by diminishing the ion concentration gradient across the membrane. These events are crucial in initiating mitochondrial apoptosis (MS et al., 2010). Increased ROS levels are known to suppress tumor growth by promoting continuous cell cycle inhibition, positioning ROS as a potential tumor suppressor (S et al., 2014). Consequently, enhancing ROS levels can effectively hinder the proliferation of hepatic carcinoma cells and initiate mitochondria-mediated apoptosis. Studies have demonstrated that the suppression of the PI3K/AKT/GSK-3β signaling pathway can increase ROS quantities, thereby yielding significant anti-liver cancer effects.

Extensive research has established that Lappaconitine (LA), an alkaloid derived from the roots of Aconitum sinomontanum Nakai, possesses a wide range of pharmacological activities. Known for its rich content of diterpene alkaloids, this plant extract has been shown to have antinociceptive, antiarrhythmic, anti-inflammatory, and analgesic properties (L et al., 2022). These attributes point to LA’s potential in various therapeutic contexts, including pain relief, cardiac rhythm regulation, inflammation reduction, and even tumor growth inhibition (Qu et al., 2019). Despite these promising properties, the specific impact and underlying mechanisms of Lappaconitine Sulfate (LS), particularly in the context of liver cancer, remain largely unexplored. This gap in knowledge presents an opportunity for future scientific inquiry.

To further explore the effect of LS on the activity of liver cancer cells, Zhang Xuemei et al. (X et al., 2020) conducted a series of *in vivo* and *in vitro* experiments to explore the effects and mechanisms of LS on HepG2 liver cancer cells. Utilizing the HepG2 xenograft tumor model, the study assessed the anti-tumor activity of LS *in vivo*. The results from these experiments provided compelling evidence that LS treatment significantly suppressed tumor weight. Histopathological analysis using H&E staining revealed a marked reduction in tumor cell population post-LS treatment, indicating its potent anti-tumor properties in nude mice with HepG2 xenograft tumors. In the *in vitro* experiments, LS was observed to induce cell cycle arrest in the G0/G1 phase and subsequently trigger apoptosis. Moreover, LS treatment led to an increase in ROS levels, which resulted in a decrease in mitochondrial membrane potential. Further molecular analysis showed a significant reduction in phosphorylation levels of key components of the PI3K/AKT/GSK-3β signaling pathway in cells treated with LS. These findings suggest that LS exerts its anti-tumor effects by simultaneously enhancing ROS generation, inhibiting the PI3K/AKT/GSK-3β pathway, and restraining HepG2 cell proliferation. This cascade of events leads to mitochondria-mediated apoptosis in liver cancer cells. The evidence strongly supports the potential of LS as an effective agent for inhibiting the activity and proliferation of liver cancer cells. Consequently, LS emerges as a promising candidate for liver cancer therapy, offering a novel approach to combat tumor growth and induce apoptosis in cancerous cells.

Curcumin, a yellow pigment extracted from the rhizome of turmeric (Liu et al., 2022), has a variety of biological effects, including anti-inflammatory、anti-oxidation、Anti-tumor, etc (FJ and SWG, 2020), because of its low toxicity and easy availability, it has broad application prospects. Despite extensive research on curcumin, a versatile and multifunctional compound, its inhibitory effects on liver cancer still lack clarity in terms of the underlying mechanisms.

In order to explore the inhibitory effect of curcumin on liver cancer cells, BaiChunhua et al. (C B. et al., 2022), investigated the inhibitory effects of Curcumin on liver cancer cells through a series of *in vivo* and *in vitro* experiments. In the *in vivo* studies, a nude mouse transplanted tumor model using HepG2 cells was established. The results demonstrated that Curcumin effectively reduced tumor volume and disrupted tumor tissue integrity in a concentration-dependent manner. *In vitro* experiments further confirmed Curcumin’s inhibitory impact on the proliferation of human liver cancer cells. The researchers observed both concentration-dependent and time-dependent inhibition of cell growth. Notably, Curcumin induced cell cycle arrest in the G0/G1 phase, effectively preventing the cells from progressing to subsequent division stages. A significant aspect of Curcumin’s effect on liver cancer cells is its induction of apoptosis. Hoechst staining analysis revealed an increase in apoptotic cell numbers with higher Curcumin concentrations. This finding underscores Curcumin’s potential as an anti-cancer agent, promoting the natural elimination of cancer cells. Moreover, Curcumin led to an upregulation in the expression levels of apoptotic proteins, particularly influencing the regulation of Bcl-2/Bax, key proteins in the mitochondrial apoptosis pathway. The treatment with Curcumin significantly reduced mitochondrial membrane potential, indicating a disruption in mitochondrial function. The study also shed light on Curcumin’s mechanism of action, revealing its ability to target the PI3K/AKT/GSK-3β signaling pathway. Curcumin was found to downregulate various molecules in this pathway, including PI3K, p-PI3K, AKT, p-AKT, GSK-3β, and p-GSK-3β. This signaling pathway is crucial for cell survival and proliferation, and its inhibition by Curcumin could contribute to suppressing liver cancer cell growth. Furthermore, using LY294002 to inhibit the PI3K/AKT/GSK-3β pathway, Curcumin enhanced the suppression of these pathway proteins and reduced the levels of Bcl-2/Bax. This suggests that Curcumin induces mitochondrial apoptosis in liver cancer cells by inhibiting the bclaf1-mediated PI3K/AKT/GSK-3β pathway, thereby exerting anti-liver cancer effects. Overall, these findings from Bai Chunhua et al.'s research highlight Curcumin’s potential as a promising therapeutic agent for liver cancer treatment. Its efficacy in inhibiting cell proliferation, inducing apoptosis, and targeting crucial signaling pathways supports its potential use in managing this aggressive cancer type. However, further research and clinical trials are necessary to fully explore and harness Curcumin’s therapeutic benefits in liver cancer treatment.

Erinacine, a bioactive compound isolated from the Hericium genus of edible mushrooms, belonging to the Hericaceae family. These mushrooms, known for their polysaccharide content, has been recognized for its potential medicinal benefits (X H. et al., 2017). Erinacine have been traditionally utilized in China and other Asian countries for the treatment of various ailments, including cancer, lymphatic disorders, and gastrointestinal diseases.

Despite the confirmed role of Erinacine in liver cancer cells, the molecular mechanism underlying its inhibitory effects on human liver cancer cell proliferation remains unclear. Zhou Lijie et al. (LJ et al., 2018) undertook a comprehensive study to elucidate the effects of Erinacine on human liver cancer, employing both *in vivo* and *in vitro* experimental approaches. The study particularly focused on Erinacine’s immunomodulatory activity. For the *in vitro* component, the research utilized hepatocellular carcinoma tissues and adjacent normal tissues from a cohort of 85 patients who underwent surgical resection for liver cancer. The study utilized immunohistochemistry to assess the expression levels of key proteins in the PI3K/Akt/GSK-3β signaling pathway. HepG-2 cells were treated with LY294002, an inhibitor of this pathway, alongside varying concentrations of Erinacine. This approach aimed to ascertain whether LY294002 influenced the effects of Erinacine on these cells. The findings revealed that both Erinacine and LY294002 induced several changes in mitochondrial function. These included a decrease in mitochondrial membrane potential, an increase in intracellular mitochondrial Ca2+ levels, and enhanced release of cytochrome C (cyt-C). Erinacine was observed to reduce mitochondrial membrane potential and downregulate the expression of proteins involved in cell signaling pathways. These alterations led to decreased cell proliferation, reduced colony formation ability, and smaller xenograft tumor sizes *in vivo*. Additionally, the expression levels of E-cadherin, Bax, and caspase-9, as well as the rate of cell apoptosis, were found to increase proportionally with the dosage of Erinacine. The study verified that Erinacine induces a dose-dependent increase in the opening of the mitochondrial permeability transition pore, achieved by suppressing the PI3K/Akt/GSK-3β signaling pathway. As a result, this promotes mitochondrial apoptosis and effectively inhibits the proliferation of liver cancer cells.

**FIGURE 3:**
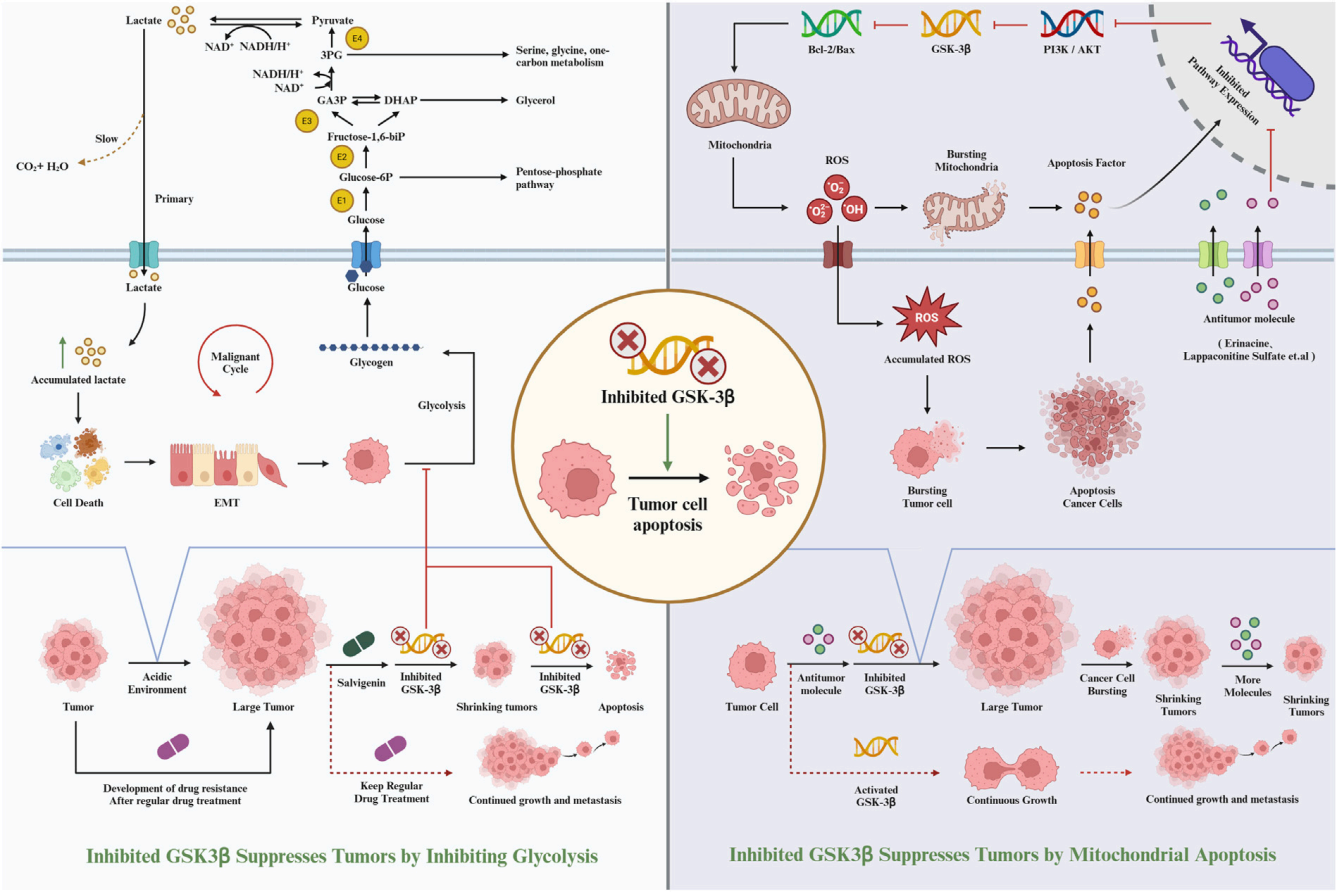
Inhibiting GSK-3β inhibits the occurrence of liver cancer.

Chonglou, a distinguished botanical resource, is extensively utilized in traditional Chinese medicine, addressing a wide array of health issues including abscesses, throat inflammations, venomous snake bites, external injuries, chronic hemorrhaging, and tumor growth (Yang et al., 2022). The monomers extracted from C. chrysanthemum, a variant of Chonglou, have demonstrated potent anticancer effects against various cancer cell types (Y et al., 2013). Recent research has focused on a specific monomer, PP-10, which has been shown to induce apoptosis and autophagy in the BGC-823 human gastric cancer cell line. This effect is achieved through the upregulation of p15.6 and the inhibition of the PI3K/Akt signaling pathway (JY et al., 2016). PP-26 is a monomer purified from Chonglou, but the anticancer activity and mechanism of purified PP-26 have not been previously described. Understanding the potential therapeutic properties and mechanisms of PP-26 could contribute to the development of novel anticancer treatments.

(Q et al., 2019) conducted studies to evaluate the efficacy of PP-26 against liver cancer, particularly focusing on its impact on HepG2 cells. Their research revealed that PP-26 inhibits the proliferation of HepG2 cells in a dose-dependent manner, primarily by inducing G2/M phase cell cycle arrest. Additionally, PP-26 was found to trigger apoptosis, a crucial cellular process in both pathological and physiological contexts, involving extrinsic and intrinsic signaling pathways (RM et al., 2015). In their experiments, HepG2 cell morphology exhibited typical apoptotic changes. This included the downregulation of apoptotic proteins such as caspase 9, caspase 3, PARP, Bcl-2, Bcl-xL, and Mcl-1, and the upregulation of Bax. Annexin V-FITC/PI double staining further confirmed apoptosis in the cells. Western blot analysis demonstrated that PP-26 effectively inhibited AKT kinase activity and consequently hindered the activation of downstream signaling pathways. This was evidenced by a reduction in both phosphorylated and total AKT levels in the cells. Moreover, after exposure to different concentrations of PP-26, a decrease in phosphorylated GSK-3β levels was observed. The study also investigated the synergistic effect of combining PP-26 with 5-FU (C et al., 2007), The MTT assay results indicated that the growth inhibition rate was higher when both 5-FU and PP-26 were used together compared to the use of 5-FU alone. This suggests that PP-26 can enhance the efficacy of 5-FU in suppressing HepG2 cell proliferation. Therefore, the combination of 5-FU and PP-26 appears to be more effective in inhibiting the growth of liver cancer cells. In summary, the findings from Qiang Li et al.'s research suggest that PP-26 has the potential to inhibit liver cancer cell proliferation. The mechanism of this inhibition involves targeting the PI3K/Akt/GSK-3β signaling pathway and promoting mitochondrial apoptosis. This positions PP-26 as a promising agent in the treatment of liver cancer, either as a standalone therapeutic or in combination with established chemotherapeutic drugs like 5-FU (Figure 3).

## 8 Therapeutic value of PI3K/AKT/GSK-3β related inhibitors in liver cancer

The therapeutic potential of targeting the PI3K/AKT/GSK-3β signaling pathway in hepatocellular carcinoma (HCC) is an area of significant interest in cancer research. The PI3K/AKT pathway plays a crucial role in regulating cell survival, growth, and proliferation. Dysregulation of this pathway is a common feature in HCC, making it an attractive target for therapeutic intervention.

Phosphoinositide 3-kinases (PI3Ks) are enzymes involved in cellular functions such as growth, proliferation, and differentiation. Selective inhibition of PI3K has shown promise in treating liver cancer. For instance, GSK2636771, a PI3Kβ inhibitor, has demonstrated efficacy in patients with advanced solid tumors, including those with PTEN-deficient HCC. Clinical trials have indicated that GSK2636771 can inhibit tumor growth and enhance the effectiveness of other therapeutic agents (Mateo et al., 2017). Additionally, BEZ235 (Dactolisib), a dual PI3K/mTOR inhibitor, has shown antitumor activity in preclinical models of HCC by targeting the PI3K/AKT/mTOR pathway, reducing tumor cell proliferation and inducing apoptosis (Oh et al., 2016).

AKT, also known as protein kinase B, is a key downstream effector of the PI3K pathway. Its activation promotes cell survival and growth, making it a critical target for cancer therapy. MK-2206, an allosteric AKT inhibitor, has demonstrated antitumor activity in various cancer models, including HCC. By inhibiting AKT phosphorylation, MK-2206 reduces cell proliferation and induces apoptosis in HCC cells (Yap et al., 2008; Hirai et al., 2010). Perifosine, another oral AKT inhibitor, has shown potential in treating HCC by interfering with the AKT signaling pathway, leading to reduced tumor viability (Richardson et al., 2012).

Glycogen synthase kinase-3 beta (GSK-3β) is a multifunctional kinase involved in various cellular processes, including metabolism, cell cycle regulation, and apoptosis. Inhibiting GSK-3β has shown promise in cancer therapy, including HCC. LY2090314, a selective GSK-3β inhibitor, has demonstrated preclinical efficacy in HCC by promoting apoptosis and inhibiting cell proliferation. This inhibitor sensitizes cancer cells to chemotherapy and targeted therapies, enhancing their effectiveness (Palomo and Martinez, 2017; Walz et al., 2017). Similarly, Tideglusib has shown antitumor activity in preclinical studies, reducing tumor growth and enhancing the effects of other anticancer agents in HCC models (del Ser et al., 2013; Martineza and Perezb, 2008).

Combining PI3K/AKT/GSK-3β inhibitors with other therapeutic agents has shown enhanced efficacy in HCC treatment. For example, combining PI3K inhibitors with checkpoint inhibitors or traditional chemotherapies can lead to synergistic effects, improving overall treatment outcomes (Yu et al., 2022).

In summary, the PI3K/AKT/GSK-3β signaling pathway is a critical regulator of cell growth, proliferation, and survival in HCC. Inhibitors targeting this pathway have shown significant therapeutic potential, both as monotherapies and in combination with other treatments. Ongoing clinical trials and preclinical studies continue to explore the full potential of these inhibitors, offering hope for more effective treatments for liver cancer.

## 9 PI3K/AKT/GSK-3β pathway inhibitors in liver cancer: Clinical research insights

In clinical research, inhibitors targeting the PI3K/AKT/GSK-3β pathway have shown significant potential in treating liver cancer.

GSK2636771, a selective PI3Kβ inhibitor, is currently being studied for its efficacy in patients with advanced solid tumors, including liver cancer. The focus of this study is to determine the recommended dose, evaluate antitumor activity, and assess safety (NIH, 2022a). Another study involves SL-901, a PI3K inhibitor, being tested in a Phase 1 clinical trial for patients with advanced solid tumors. The primary objectives are to identify the maximum tolerated dose, optimal dosing regimen, pharmacokinetics, and preliminary efficacy, which are significant for liver cancer therapy (NIH, 2022b). Additionally, a Phase 2 study of Copanlisib combined with Fulvestrant is ongoing for patients with ER+ and/or PR + cancers with PI3K and PTEN alterations. Although primarily focusing on breast and endometrial cancers, the results could provide valuable insights for liver cancer treatment strategies (NIH, 2021).

Vevorisertib (ARQ 751) is an AKT inhibitor currently being investigated either as a single agent or in combination with other anticancer agents in patients with solid tumors having PIK3CA, AKT, or PTEN mutations. This study aims to determine the safety, tolerability, and preliminary efficacy of the drug in advanced solid tumors, including liver cancer (NIH, 2016). Another AKT inhibitor, MK2206, is being evaluated for its efficacy in treating advanced breast cancer and solid tumors, including liver cancer. The study includes assessments of tumor response and the pharmacodynamics of MK2206 (NIH, 2011).

9-ING-41 is a GSK-3β inhibitor being tested in a Phase 1/2 clinical trial as a single agent and combined with chemotherapy in patients with refractory hematologic malignancies or solid tumors, including liver cancer. The study aims to assess the safety, tolerability, and preliminary antitumor activity of 9-ING-41 (NIH. 9-ING-41, 2018).

These studies demonstrate the significant potential of inhibitors targeting the PI3K/AKT/GSK-3β pathway in treating liver cancer and other advanced solid tumors. Ongoing clinical trials and research continue to explore the full potential of these inhibitors, aiming to improve outcomes for patients with challenging malignancies, thus supporting the therapeutic value of this approach in clinical oncology research.

## 10 Effect of PI3K/AKT/GSK-3β on drug resistance of liver cancer

Despite the significant progress made in chemotherapy, which has resulted in a decrease in mortality rates among cancer patients, the overall survival rates after 5 years still remain disappointingly low. This can be primarily attributed to the presence of inherent or acquired resistance mechanisms to the antineoplastic drugs used in treatment. These resistance mechanisms undermine the effectiveness of chemotherapy and pose a major challenge in improving the prognosis for cancer patients. Consequently, there is a need for further research and understanding of these resistance mechanisms in order to develop more effective treatment strategies and improve patient outcomes (A, 2017). Tumor multidrug resistance (MDR) is a significant factor contributing to the inadequate efficacy of tumor treatments. This resistance is frequently linked with the expression of PI3K, a class of lipid kinases that are pivotal in regulating a multitude of physiological processes. In the context of tumor cells, the expression and activity of PI3K are closely associated with the development of drug resistance. A critical component within the PI3K pathway is AKT, serving as an essential effector molecule. AKT plays a vital role in modulating cell apoptosis, stimulating cell growth, and regulating cellular energy metabolism. Abnormal activation of the PI3K/AKT signaling pathway has been identified as a significant contributing factor to the phenomenon of multidrug resistance in tumors. This pathway, when dysregulated, can confer survival advantages to tumor cells, allowing them to resist the cytotoxic effects of various chemotherapeutic agents. The aberrant activation leads to enhanced cell survival, proliferation, and altered metabolic processes, all of which contribute to the cells’ ability to withstand anti-cancer drug. The challenge posed by tumor MDR is intricately linked to the expression and abnormal activation of the PI3K/AKT signaling pathway (Li et al., 2020). It causes cellular multidrug resistance by activating the PI3K/AKT signaling pathway.

### 10.1 The PI3K/AKT pathway participates in the regulation of aerobic glycolysis to increase the energy supply of MDR

The activation of the PI3K/AKT pathway is initiated by the generation of phosphoinositides, specifically phosphorylated at the 3′position. This signaling pathway is critically implicated in the development of MDR across a diverse spectrum of cancers, including breast cancer, leukemia, lung cancer, ovarian cancer, hepatocellular carcinoma, and melanoma (D et al., 2019). The activation of the PI3K/AKT pathway plays a key role in conferring the MDR phenotype to cancer cells, which is characterized by their resistance to cytotoxic anticancer drugs.

In the development of tumors, energy metabolism plays a pivotal role, markedly differentiating tumor cells from their normal counterparts (YC et al., 2020). One key aspect of this differentiation is the ability of tumor cells to undergo metabolic reprogramming during chemotherapy treatment. This reprogramming allows tumor cells to adapt their energy conversion processes, which in turn enables them to develop resistance to anti-cancer drugs (V et al., 2017). Among the various distinctive characteristics of these metabolic changes, the Warburg effect is particularly notable. Through this effect, tumor cells primarily rely on glycolysis for energy production and biosynthetic processes, even in the presence of oxygen. The PI3K/AKT signaling pathway plays a crucial role in governing aerobic glycolysis and the central metabolism of glucose in tumor cells (RB and N, 2009). Alterations in the intracellular microenvironment, as a result of these metabolic changes, lead to an increase in MDR expression, consequently affecting the efficacy of tumor treatments. For instance, in K562/ADM cells, a doxorubicin-resistant human chronic myeloid leukemia cell line, the activation of the PI3K/AKT signaling pathway is notably prominent. This activation stimulates aerobic glycolysis and enhances the production of polypeptides within tumor cells, contributing to the development of drug resistance (X Z. et al., 2018).

The glycolytic process in tumor cells is intricately controlled by the PI3K/AKT signaling pathway, which plays a crucial role in upregulating proteins involved in glucose metabolism, such as glucose transporters (GLUT) and phosphofructokinase (PFK) (W et al., 2019). GLUT1, a key glucose transporter, is particularly significant in the transport of glucose across different tumor types. The activation of the PI3K/AKT pathway effectively enhances the expression of GLUT1, facilitating the increased glucose uptake necessary to meet the high metabolic demands of rapidly proliferating tumor cells (C et al., 2018). This heightened expression of GLUT1 promotes enhanced glucose metabolism within cancer cells. This is evident in MDR cells, where an augmented capacity to rapidly transport and utilize glucose through glycolysis is observed, leading to increased ATP production and supporting the proliferation of neoplastic cells (X Z. et al., 2017). Additionally, PI3K can directly phosphorylate and activate PFK2 via AKT, increasing the synthesis of fructose-2,6-bisphosphate, which in turn activates PFK1, the rate-limiting enzyme of glycolysis. Consequently, this activation leads to an increase in the glycolytic flux (JH et al., 2018). Further research has indicated that inhibiting glycolysis can disrupt ATP production and reverse drug resistance mediated by P-glycoprotein (P-gp). From these findings, it can be inferred that the PI3K/AKT signaling pathway plays a pivotal role in controlling aerobic glycolysis and holds a critical regulatory function in tumor MDR. Additionally, this pathway acts as a key energy source for tumor cells, particularly those combatting the effects of multiple drugs.

### 10.2 PI3K/AKT phosphorylates GSK-3β to promote MDR-associated tumor proliferation

GSK-3β is a serine/threonine kinase, is a crucial regulator of cell proliferation in response to various stimuli. It has been observed to be abnormally overexpressed in a variety of malignant tumors, including colon cancer, liver cancer, ovarian cancer, and pancreatic cancer. The dysregulation of GSK-3β is closely associated with the development of these cancers (M et al., 2020).

In the context of cellular signaling, GSK-3β plays a pivotal role, particularly within the PI3K/AKT pathway. It is primarily regulated by AKT, which phosphorylates GSK-3β, rendering it inactive. However, it has been discovered that the activity of GSK-3β can also be modulated through the phosphorylation of other residues, leading to enhanced activation of AKT (128). One of the notable effects of AKT activation is the decreased expression of E-cadherin. Since β-catenin is associated with E-cadherin at the cell membrane, the loss of E-cadherin leads to an increase in cytoplasmic β-catenin. Conversely, when GSK-3β is phosphorylated or inactivated, β-catenin accumulates in the cytoplasm and then translocates into the nucleus. In the nucleus, β-catenin interacts with various cytokines, such as lymphokines, and accelerates transcription processes. This results in increased expression of oncogenes such as c-Jun, c-Myc, and cyclin D1, thereby promoting tumor proliferation (M et al., 2019). Furthermore, the aberrant activation of GSK-3β enhances the expression of target genes like MDR1 and survivin. This upregulation plays a critical role in the development of MDR in tumors. By influencing gene expression related to cell survival and drug efflux, GSK-3β contributes to the ability of cancer cells to resist chemotherapy (S et al., 2017).

To summarize, the interaction between GSK-3β and the PI3K/AKT pathway controls the growth and programmed cell death of tumors, thereby influencing the occurrence of MDR.

### 10.3 Combination of vinpocetine and sorafenib improves sorafenib resistance

Chemoembolization and multikinase inhibitors are key modalities in the current therapeutic landscape for treating patients with intermediate and advanced HCC. Sorafenib, an orally administered multikinase inhibitor, has gained approval from the FDA for the treatment of advanced HCC. It targets several critical kinase sites, including Raf-1, B-Raf, and the receptors for vascular endothelial growth factor and fibroblast growth factor-β (YS et al., 2007). While Sorafenib has demonstrated efficacy in prolonging the survival of liver cancer patients by approximately 3–5 months, a major limitation of this therapy is the eventual development of acquired drug resistance (YJ et al., 2017). This resistance significantly diminishes the drug’s effectiveness over time. Patients who develop resistance to Sorafenib often experience a relapse in tumor growth. Consequently, the search for new treatments for patients who have acquired resistance to Sorafenib is of paramount importance.

Vinpocetine, derived from the seeds of Catharanthus roseus, is primarily recognized for its use in treating cerebrovascular diseases such as stroke, cerebral hemorrhage, and cognitive dysfunction (YS et al., 2018). It functions as a phosphodiesterase type 1 (PDE-1) inhibitor, relaxing brain smooth muscle cells, enhancing cerebral blood flow, and exhibiting anti-inflammatory and anti-obesity effects (NJ et al., 2019; K et al., 2020). Additionally, Vinpocetine’s anticancer properties have been attributed to its phosphodiesterase-inhibiting mechanism, particularly in inhibiting the growth of glioblastoma multiforme cells by targeting PDE-1. It has also been observed to modulate the AKT signaling pathway in breast cancer cell lines (EW et al., 2012), significantly reducing tumor growth and causing cell cycle arrest in the G0/G1 phase.

Zhang Zuoyan et al. (ZY et al., 2021) conducted a study to explore the cytotoxic synergistic mechanism of Vinpocetine in combination with Sorafenib in liver cancer cells and to investigate the relevant signaling pathways involved. Their research revealed that while Sorafenib alone activated regulators of the PI3K pathway, its combination with Vinpocetine effectively blocked this pathway. This blockade resulted in the activation of key components such as PI3K, phospho-mTOR, and phospho-AKT, leading to a reduction in Sorafenib-resistant HCC cells. These findings suggest that resistance to Sorafenib is mediated by the activation of the PI3K/AKT signaling pathway, and inhibiting this pathway can reverse resistance in HCC cells (YJ et al., 2017; H et al., 2018). The study demonstrated that the combination of Vinpocetine and Sorafenib inhibits the PI3K/AKT pathway, indicating its potential effectiveness in treating liver cancer patients resistant to Sorafenib. Furthermore, AKT-mediated phosphorylation of Ser-9 inhibits GSK-3β, resulting in a rapid decrease in cellular ATP production and the initiation of autophagy in cancer cells (A et al., 2016).

In summary, Vinpocetine targets the PI3K/AKT/GSK-3β signaling pathway to suppress liver cancer cell growth. It induces autophagy for cell protection and enhances the susceptibility of liver cancer cells to Sorafenib therapy. This sensitization effectively reduces the resistance of liver cancer cells to Sorafenib, suggesting a promising combination therapy for liver cancer.

## 11 Potential therapeutic mechanisms targeting PI3K/AKT/GSK-3β in liver cancer

### 11.1 Inhibition of PI3K/AKT pathway components

#### 11.1.1 1PI3K inhibitors

GSK2636771: Selective PI3K inhibitor, shows efficacy in PTEN-deficient HCC.

BEZ235 (Dactolisib): Dual PI3K/mTOR inhibitor, reduces tumor cell proliferation and induces apoptosis.

#### 11.1.2 2AKT inhibitors

MK-2206: Allosteric AKT inhibitor, reduces cell proliferation and induces apoptosis.

Perifosine: Oral AKT inhibitor, interferes with AKT signaling and reduces tumor viability.

#### 11.1.3 3GSK-3β inhibitors

LY2090314: Selective GSK-3β inhibitor, promotes apoptosis and inhibits cell proliferation.

Tideglusib: Shows antitumor activity, reduces tumor growth, and enhances the effects of other anticancer agents.

### 11.2 Combination therapies

Vinpocetine and Sorafenib: Combination inhibits the PI3K/AKT pathway, reducing Sorafenib-resistant HCC cells by blocking key components such as PI3K, phospho-mTOR, and phospho-AKT.

### 11.3 Targeting glycolysis and metabolic pathways

Salvigenin: Inhibits glycolysis by reducing glucose absorption and lactate production, leading to decreased levels of key glycolytic enzymes. It impedes the phosphorylation of GSK-3β by inhibiting the PI3K/AKT pathway.

Dezocine: Modulates the Akt1-GSK-3β pathway, altering glycolytic enzyme activity, and affecting the viability and migration of HepG2 cells.

Curcumin: Downregulates PI3K, p-PI3K, AKT, p-AKT, GSK-3β, and p-GSK-3β, induces mitochondrial apoptosis by inhibiting the bclaf1-mediated PI3K/AKT/GSK-3β pathway.

Erinacine: Induces mitochondrial apoptosis and reduces cell proliferation by suppressing the PI3K/Akt/GSK-3β signaling pathway.

PP-26: Inhibits the PI3K/Akt/GSK-3β signaling pathway and promotes mitochondrial apoptosis, enhancing the efficacy of 5-FU in inhibiting HepG2 cell proliferation.

### 11.4 Modulation of tumor microenvironment and immune response

Clusterin (sCLU): Activates the AKT/GSK-3β/β-catenin signaling pathway, contributing to chemotherapy resistance, metastasis, and poor prognosis in HCC.

Peroxisome Proliferator-Activated Receptor δ (PPARδ): Promotes proliferation, migration, and angiogenesis by activating the PI3K/AKT/GSK-3β signaling pathway.

Programmed Cell Death 6 (PDCD6): Enhances proliferation, migration, and invasion capabilities of liver cancer cells via activation of the AKT pathway, leading to GSK-3β phosphorylation.

### 11.5 Non-coding RNAs (ncRNAs) regulation

miRNAs and lncRNAs: Modulate the PI3K/AKT/GSK-3β pathway by targeting key components and influencing gene expression related to tumor growth and resistance. Examples include miR-96, miR-21, miR-221, miR-222, and HULC.

Targeting the PI3K/AKT/GSK-3β signaling pathway presents multiple therapeutic mechanisms for combating liver cancer, including inhibition of key pathway components, combination therapies, targeting glycolysis and metabolic pathways, modulating the tumor microenvironment, and regulating gene expression through non-coding RNAs. These approaches hold promise for improving treatment outcomes and overcoming resistance to existing therapies in liver cancer patients.

## 12 VOSviewer

**FIGURE 4:**
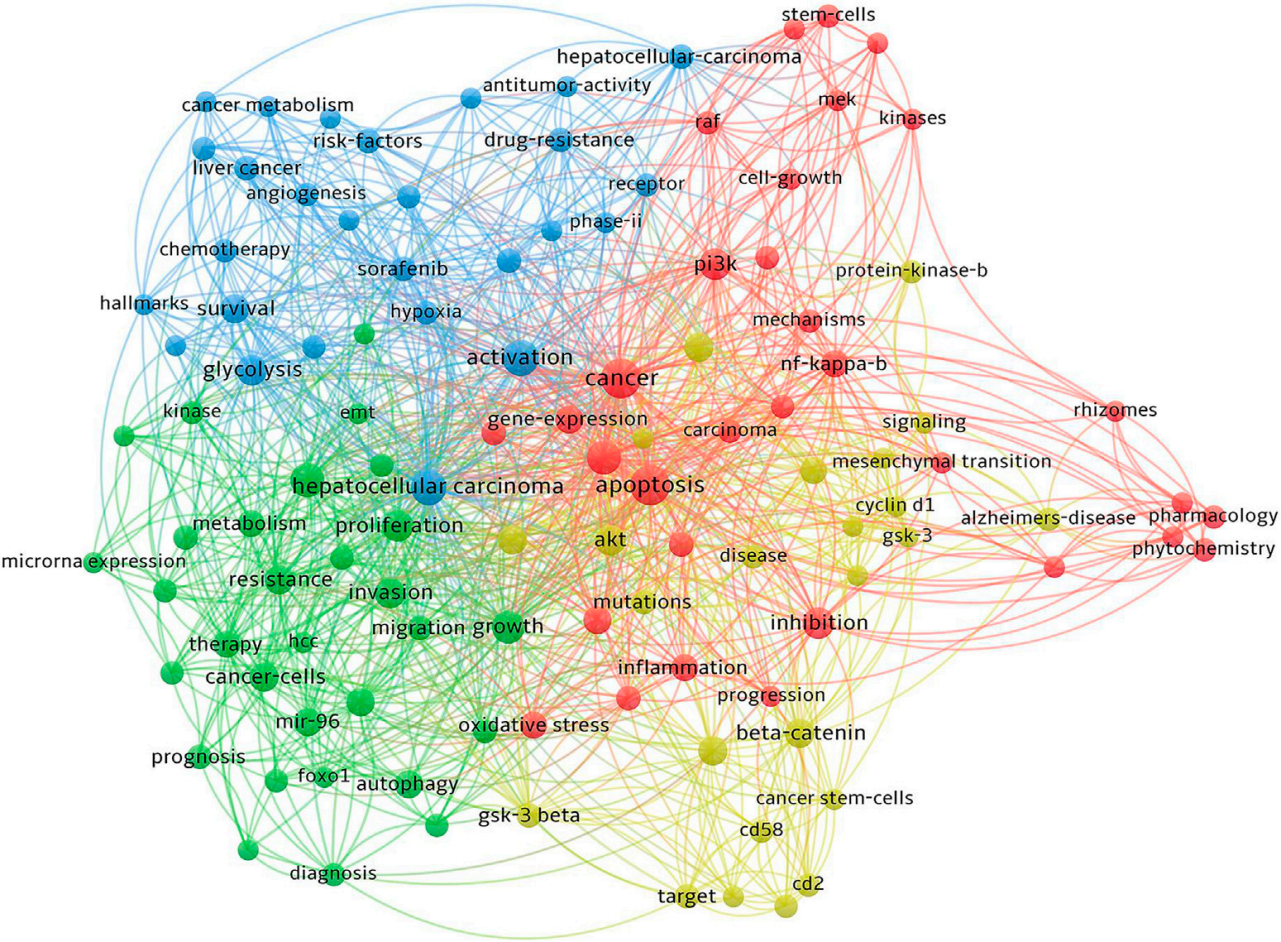
VOSviewer_PI3KAKT.

We used VOSviewer to create a figure highlighting evaluation of the effect of GSK-3β on liver cancer based on the PI3K/AKT pathway themes, activities, and sources to enhance the review methodology. Each node represents a keyword, with node size indicating the keyword’s importance within the literature. The lines connecting nodes represent co-occurrence relationships between keywords. Keywords are color-coded based on the clusters they belong to, where keywords in the same cluster are more likely to co-occur in the literature (Figure 4).

## 13 Outlook

Hepatocellular carcinoma (HCC), a primary malignancy of liver cells, is recognized globally as the third leading cause of cancer-related deaths, primarily due to its highly invasive nature. The current frontline treatment for advanced HCC includes targeted therapies such as sorafenib and lenvatinib, which are anti-angiogenic and anti-proliferative kinase inhibitors, along with immunotherapeutic approaches like the combination of atezolizumab and bevacizumab. However, kinase inhibitors have been shown to provide only a modest extension in survival, typically by a few months, while immunotherapy is effective only in a specific subset of liver cancer patients. Recent years have seen a growing acceptance of combination therapies and compounds derived from traditional Chinese medicine in the treatment of liver cancer. These approaches have demonstrated potential in reducing the toxicity associated with chemotherapy, improving patient prognosis, and combating resistance to targeted liver cancer drugs. As such, the future direction in liver cancer treatment is increasingly focused on understanding the pathogenesis of HCC and developing more effective drug combinations.

A key factor in the onset and progression of liver cancer is the PI3K/AKT/GSK-3β signaling pathway. This pathway plays a significant role in the growth, proliferation, metastasis, and apoptosis of liver cancer cells. It is also implicated in the development of resistance to chemotherapy in liver cancer, highlighting its importance in therapeutic strategies. Studies have shown that inhibiting the PI3K/AKT/GSK-3β pathway can reduce aerobic glycolysis in liver cancer cells and promote apoptosis. This inhibition increases reactive oxygen species (ROS) levels, leading to damage in the mitochondrial membrane, changes in membrane permeability, a decrease in mitochondrial membrane potential, and the initiation of mitochondrial apoptosis, thereby impeding the progression of liver cancer cells. GSK-3β, in particular, has been identified as both a prognostic indicator in clinical treatment and a target for suppressing tumor growth and metastasis. Additionally, it plays a role in enhancing resistance to chemotherapy and radiotherapy. As such, clinical studies focusing on the influence of GSK-3β on the PI3K/AKT pathway show promise in the treatment of liver cancer.

In summary, the evolving landscape of HCC treatment underscores the importance of targeting the PI3K/AKT/GSK-3β pathway. The development of therapies that can effectively modulate this pathway may offer new avenues for improving outcomes in liver cancer patients, particularly in overcoming drug resistance and enhancing the efficacy of existing treatments.
